# Supplementary material for: Pyruvate Dehydrogenase Kinase 1 inhibition mediated oxidative phosphorylation enhancement in cartilage promotes osteoarthritis progression
Source: BMC Musculoskelet Disord. 2023 Jul 20;24:597. doi: 10.1186/s12891-023-06585-6 (PMC10357736; doi:10.1186/s12891-023-06585-6)
Supplement: Supplementary file 1 — Additional file 1. [file 12891_2023_6585_MOESM1_ESM.zip › MF.Gsea.1653627316242/GOMF_STRUCTURAL_CONSTITUENT_OF_MUSCLE.html]

Details for gene set GOMF\_STRUCTURAL\_CONSTITUENT\_OF\_MUSCLE[GSEA]

|  || Dataset | 55457\_collapsed\_to\_symbols.cls.cls #OA\_versus\_normal.cls.cls #OA\_versus\_normal\_repos |
| Phenotype | cls.cls#OA\_versus\_normal\_repos |
| Upregulated in class | OA |
| GeneSet | GOMF\_STRUCTURAL\_CONSTITUENT\_OF\_MUSCLE |
| Enrichment Score (ES) | 0.4186835 |
| Normalized Enrichment Score (NES) | 1.6268167 |
| Nominal p-value | 0.012048192 |
| FDR q-value | 0.1326194 |
| FWER p-Value | 0.968 |
Table: GSEA Results Summary

  

Fig 1: Enrichment plot: GOMF\_STRUCTURAL\_CONSTITUENT\_OF\_MUSCLE      
 Profile of the Running ES Score & Positions of GeneSet Members on the Rank Ordered List

  

| SYMBOL | TITLE | RANK IN GENE LIST | RANK METRIC SCORE | RUNNING ES | CORE ENRICHMENT || 1 | MYL6B | myosin light chain 6B [Source:HGNC Symbol;Acc:HGNC:29823] | 815 | 0.565 | -0.0042 | Yes |
| 2 | SORBS2 | sorbin and SH3 domain containing 2 [Source:HGNC Symbol;Acc:HGNC:24098] | 1707 | 0.397 | -0.0319 | Yes |
| 3 | MYOM2 | myomesin 2 [Source:HGNC Symbol;Acc:HGNC:7614] | 1910 | 0.372 | -0.0087 | Yes |
| 4 | KRT19 | keratin 19 [Source:HGNC Symbol;Acc:HGNC:6436] | 2258 | 0.331 | -0.0009 | Yes |
| 5 | MYBPC1 | myosin binding protein C1 [Source:HGNC Symbol;Acc:HGNC:7549] | 2364 | 0.319 | 0.0243 | Yes |
| 6 | MYL2 | myosin light chain 2 [Source:HGNC Symbol;Acc:HGNC:7583] | 2376 | 0.317 | 0.0567 | Yes |
| 7 | MYBPC2 | myosin binding protein C2 [Source:HGNC Symbol;Acc:HGNC:7550] | 2393 | 0.315 | 0.0885 | Yes |
| 8 | NEB | nebulin [Source:HGNC Symbol;Acc:HGNC:7720] | 2395 | 0.315 | 0.1214 | Yes |
| 9 | CSRP3 | cysteine and glycine rich protein 3 [Source:HGNC Symbol;Acc:HGNC:2472] | 2411 | 0.313 | 0.1530 | Yes |
| 10 | MYL1 | myosin light chain 1 [Source:HGNC Symbol;Acc:HGNC:7582] | 2491 | 0.303 | 0.1787 | Yes |
| 11 | TTN | titin [Source:HGNC Symbol;Acc:HGNC:12403] | 2492 | 0.303 | 0.2105 | Yes |
| 12 | ACTN2 | actinin alpha 2 [Source:HGNC Symbol;Acc:HGNC:164] | 2532 | 0.299 | 0.2388 | Yes |
| 13 | CSRP1 | cysteine and glycine rich protein 1 [Source:HGNC Symbol;Acc:HGNC:2469] | 2585 | 0.292 | 0.2654 | Yes |
| 14 | MYL5 | myosin light chain 5 [Source:HGNC Symbol;Acc:HGNC:7586] | 2705 | 0.279 | 0.2854 | Yes |
| 15 | MYLPF | "myosin light chain, phosphorylatable, fast skeletal muscle [Source:HGNC Symbol;Acc:HGNC:29824]" | 2816 | 0.268 | 0.3050 | Yes |
| 16 | MYOT | myotilin [Source:HGNC Symbol;Acc:HGNC:12399] | 2858 | 0.265 | 0.3295 | Yes |
| 17 | CSRP2 | cysteine and glycine rich protein 2 [Source:HGNC Symbol;Acc:HGNC:2470] | 2960 | 0.254 | 0.3483 | Yes |
| 18 | MYH8 | myosin heavy chain 8 [Source:HGNC Symbol;Acc:HGNC:7578] | 3119 | 0.238 | 0.3609 | Yes |
| 19 | DAG1 | dystroglycan 1 [Source:HGNC Symbol;Acc:HGNC:2666] | 3203 | 0.230 | 0.3786 | Yes |
| 20 | MYBPH | myosin binding protein H [Source:HGNC Symbol;Acc:HGNC:7552] | 3310 | 0.220 | 0.3934 | Yes |
| 21 | TCAP | titin-cap [Source:HGNC Symbol;Acc:HGNC:11610] | 3490 | 0.203 | 0.4008 | Yes |
| 22 | MYL3 | myosin light chain 3 [Source:HGNC Symbol;Acc:HGNC:7584] | 3529 | 0.199 | 0.4187 | Yes |
| 23 | MYBPC3 | myosin binding protein C3 [Source:HGNC Symbol;Acc:HGNC:7551] | 3926 | 0.162 | 0.4049 | No |
| 24 | MYOM1 | myomesin 1 [Source:HGNC Symbol;Acc:HGNC:7613] | 4027 | 0.153 | 0.4132 | No |
| 25 | TPM1 | tropomyosin 1 [Source:HGNC Symbol;Acc:HGNC:12010] | 4220 | 0.135 | 0.4124 | No |
| 26 | MYL9 | myosin light chain 9 [Source:HGNC Symbol;Acc:HGNC:15754] | 4939 | 0.077 | 0.3647 | No |
| 27 | MYH11 | myosin heavy chain 11 [Source:HGNC Symbol;Acc:HGNC:7569] | 5211 | 0.056 | 0.3495 | No |
| 28 | OBSCN | "obscurin, cytoskeletal calmodulin and titin-interacting RhoGEF [Source:HGNC Symbol;Acc:HGNC:15719]" | 5230 | 0.054 | 0.3537 | No |
| 29 | SYNM | synemin [Source:HGNC Symbol;Acc:HGNC:24466] | 5300 | 0.048 | 0.3533 | No |
| 30 | DMD | dystrophin [Source:HGNC Symbol;Acc:HGNC:2928] | 5605 | 0.023 | 0.3322 | No |
| 31 | NEBL | nebulette [Source:HGNC Symbol;Acc:HGNC:16932] | 6177 | -0.019 | 0.2897 | No |
| 32 | ANKRD2 | ankyrin repeat domain 2 [Source:HGNC Symbol;Acc:HGNC:495] | 6186 | -0.019 | 0.2911 | No |
| 33 | ASPH | aspartate beta-hydroxylase [Source:HGNC Symbol;Acc:HGNC:757] | 7533 | -0.121 | 0.1992 | No |
| 34 | ACTN3 | actinin alpha 3 [Source:HGNC Symbol;Acc:HGNC:165] | 7557 | -0.124 | 0.2103 | No |
| 35 | TPM4 | tropomyosin 4 [Source:HGNC Symbol;Acc:HGNC:12013] | 7974 | -0.153 | 0.1940 | No |
| 36 | TPM2 | tropomyosin 2 [Source:HGNC Symbol;Acc:HGNC:12011] | 8156 | -0.167 | 0.1975 | No |
| 37 | MYL6 | myosin light chain 6 [Source:HGNC Symbol;Acc:HGNC:7587] | 9188 | -0.247 | 0.1431 | No |
| 38 | CAPN3 | calpain 3 [Source:HGNC Symbol;Acc:HGNC:1480] | 9326 | -0.258 | 0.1595 | No |
| 39 | PLEC | plectin [Source:HGNC Symbol;Acc:HGNC:9069] | 10799 | -0.405 | 0.0875 | No |
| 40 | SMTN | smoothelin [Source:HGNC Symbol;Acc:HGNC:11126] | 12447 | -0.723 | 0.0352 | No |
Table: GSEA details [plain text format]

  

Fig 2: GOMF\_STRUCTURAL\_CONSTITUENT\_OF\_MUSCLE      
 Blue-Pink O' Gram in the Space of the Analyzed GeneSet

  

Fig 3: GOMF\_STRUCTURAL\_CONSTITUENT\_OF\_MUSCLE: Random ES distribution      
 Gene set null distribution of ES for **GOMF\_STRUCTURAL\_CONSTITUENT\_OF\_MUSCLE**
